# Differing specificities and isotypes of anti-citrullinated peptide/protein antibodies in palindromic rheumatism and rheumatoid arthritis

**DOI:** 10.1186/s13075-017-1329-6

**Published:** 2017-06-15

**Authors:** Sonia Cabrera-Villalba, María José Gomara, Juan D. Cañete, Julio Ramírez, Georgina Salvador, Virginia Ruiz-Esquide, Maria Victoria Hernández, José Inciarte-Mundo, Isabel Haro, Raimon Sanmartí

**Affiliations:** 1 Unidad de Artritis, Servicio de Reumatología Hospital Clínic de Barcelona, Villarroel 170, 08036 Barcelona, Spain; 2 Unidad de Síntesis y Aplicaciones Biomédicas de Péptidos, IQAC-CSIC, Barcelona, Spain; 3 Hospital Universitario Mútua Terrassa, Terrassa, Barcelona, Spain

**Keywords:** Palindromic rheumatism, Rheumatoid arthritis, Anticitrullinated peptide/protein antibodies, Vimentin

## Abstract

**Background:**

To analyze differences in the recognition of anti-citrullinated peptide/protein antibody (ACPA) citrullinated epitopes and isotypes in patients with palindromic rheumatism (PR) and rheumatoid arthritis (RA).

**Methods:**

ACPA fine specificities (citrullinated peptides of enolase, fibrin, and vimentin) and isotypes (IgG, IgM, and IgA) were analyzed in 54 patients with longstanding PR and 54 patients with established RA.

**Results:**

CCP2 tested positive in 66.7% of patients with PR and RA. The ACPA distribution of fine specificities and isotypes differed between PR and RA patients. PR patients had a lower frequency of fine ACPA specificities than RA patients, which was significant in the case of a peptide derived from vimentin (PR 24.1% vs. 59.3% RA; *p* < 0.001). The mean number of ACPA specificities was lower in PR than in RA patients, and only 25.9% of PR patients recognized ≥2 additional specificities compared with 46.3% of RA patients. Significantly less isotype usage, especially IgA, was observed in PR patients.

**Conclusion:**

The ACPA immune response differed in patients with PR and RA, with fewer fine specificities and isotype usage in patients with PR. Some patients with PR may have impaired maturation of the B-cell response against citrullinated peptides with no progression to RA.

## Background

Palindromic rheumatism (PR), an intermittent form of arthritis, may, in some cases, evolve to rheumatoid arthritis (RA) [1]. A considerable proportion of PR patients have the characteristic autoantibodies found in RA: rheumatoid factor (RF) and/or anti-citrullinated peptide/protein antibodies (ACPA). Our first description of the high frequency of ACPA in the sera of PR patients [2] has been confirmed by subsequent studies [3–5]. The ACPA reactivities observed in serum of patients with PR are citrulline-dependent as occurs in RA [6]. ACPA positivity in the early phases of PR has been associated with a high risk of evolution to RA [3]. However, a significant proportion of patients with PR do not evolve to RA in the long-term, even when ACPA are positive [7]. It remains unclear whether PR is a separate disease entity, an incomplete expression of RA, or a preclinical stage of RA [8].

ACPA may precede the onset of clinical RA by several years. Studies show that ACPA may be directed against several citrullinated peptides, with epitope spreading occurring over several years prior to RA onset [9]. ACPA fine specificities remain relatively stable after the definitive clinical diagnosis of RA [10]. The isotype usage of ACPA may differ in the disease phases, with less isotype usage in the preclinical stage [11, 12].

We hypothesized that ACPA responses may differ between patients with PR and RA. The ACPA immune response in PR would be closer to that observed in healthy subjects or in the preclinical phase of RA, and this might explain the absence of progression to RA in some PR patients. This study analyzed the differences in ACPA fine specificities and isotype usage in patients with longstanding PR who did not evolve to RA and in patients with established RA.

## Methods

### Study populations

This cross-sectional study included patients diagnosed with PR according to the criteria of Guerne and Weisman [1] attending our Arthritis Unit from June 2012 to June 2013, with no evolution to RA or other rheumatic diseases at the time of clinical and laboratory assessments; other causes of intermittent arthritis were excluded. As controls, we included patients with established RA (1987 ACR criteria) controlled by gender, age (±5 years), disease duration (±3 years), and CCP2 positivity. Written consent was obtained from all participants and the study was approved by the Hospital Clinic Ethics Committee.

### CCP2 assay and measurement of ACPA fine specificities and isotypes

IgG antiCCP2 was measured in serum samples by a second-generation enzyme-linked immunosorbent assay (ELISA; Immunoscan, Eurodiagnostica,; NV <50 UI). The citrullinated peptides used in this study, whose primary sequences are detailed below, are derived from the main protein targets of RA-specific autoantibodies: fibrin [13], vimentin [14], and alpha-enolase [15].

ACPA fine specificities were determined by home-made ELISA tests using synthetic citrullinated peptides previously studied by our group [16, 17] as antigens: fibrin ([Cit^630^]α-Fibrin(617-631)), vimentin ([Cit^71^]Vim(47-72) and [Cit^64,69,71^]Vim(47-72)), and α-enolase [Cys^4,22^,Cit^9,15^]α-Enolase(5-21); namely p18, p48, p55 and CEP-1, respectively. Uncitrullinated peptides were also synthesized and used as a control for citrulline specificity of the anti-citrulline peptide antibodies in both RA and PR patients. The ACPA isotypes IgG, IgA, and IgM of fine specificities were measured using the corresponding secondary antibodies (Jackson Immunoresearch) according to the previously described ELISA procedure [16, 17]. Cut-off values for each ELISA test were determined using receiver operating characteristic (ROC) curves, with a specificity of 98% compared with a healthy population (blood donors, *n* = 64).

### Peptide synthesis

Citrullinated peptides [Cit^630^]α-fibrin(617-631): HSTKRGHAKSRPVCitG (p18), [Cit^71^]Vim(47-72): STSRSLYASSPGGVYATRSSAVRLCitS (p48), [Cit^64,69,71^]Vim(47-72): STSRSLYASSPGGVYATCitSSAVCitLCitS (p55), and [Cys^4,22^,Cit^9,15^]Enolase(5-21): CKIHACitEIFDSCitGNPTVEC (CEP-1), and uncitrullinated versions α-fibrin(617-631): HSTKRGHAKSRPVRG, Vim(47-72): STSRSLYASSPGGVYATRSSAVRLRS, and Enolase(5-21): CKIHAREIFDSRGNPTVEC were synthesized by solid-phase peptide synthesis as C-terminal carboxamides on a Novasyn TGR resin (Novabiochem Merck, Germany) (0.22 meq/g) and following a 9-fluorenylmethoxycarbonyl (Fmoc) strategy. Couplings were performed by 2-(1H-7-azabenzotriazole-1-yl)-1,1,3,3-tetramethyluronium hexafluorophosphate (HATU) and diisopropylethylamine (DIEA) activation, with three-fold molar excesses of amino acids. The Fmoc deprotection step was performed twice with 20% piperidine in dimethylformamide (DMF) for 10 min. The peptides were concomitantly side chain-deprotected and cleaved from the resin by treatment with a mixture of TFA in the presence of triisopropylsilane (TIS) and water as scavengers (TFA:TIS:H_2_O, 9.5:2.5:2.5) for 3 h with occasional agitation at room temperature. The solvent was removed in vacuum and the crude peptides were precipitated with diethyl ether. The solids were dissolved in 30% acetic acid in water and lyophilized.

Crude peptides were purified by semipreparative HPLC (1260 Infinity, Agilent Technologies) in an XBridge™ Prep BEH130 C18 column (Waters, 5 μm, 10 × 250 mm) at a flow rate of 3.5 mL/min. Final peptides were characterized by analytical HPLC on a 1260 Infinity chromatograph (Agilent Technologies) with an Eclipse Plus C18 column (Agilent, 3.5 μm, 4.6 × 100 mm). The peptides were 95% pure by analytical HPLC at 220 nm. Their identity was confirmed by electrospray ionization mass spectrometry (ESI-MS) performed with a liquid chromatograph–time of flight (LC-TOF) detector, LCT Premier XE (Micromass Waters) coupled to Analytical Ultra Performance Liquid Chromatography apparatus (UPLC, Waters).

For cyclization of CEP-1, its linear version was dissolved in 0.1 M ammonium bicarbonate (0.3 mg/mL). The solution was left to stand open to the atmosphere and stirred for 24 h. The formation of cyclic disulfide was checked by the Ellman test.

### ELISA assays

Peptide sequences were coupled covalently to ELISA microplates (Costar Corp., DNA-bind N-oxysuccinimide surface, Cambridge, MA, USA). Peptides were diluted to 10 μg/mL in 0.05 M carbonate/bicarbonate (pH 9.6) buffer; 100 μL of peptide solution was added to each well of microplates and incubated overnight at 4 °C. Each plate contained control wells that included all reagents except the serum sample in order to estimate the background reading and control wells that included all reagents except the peptide to evaluate nonspecific reactions of sera. For blank controls, wells were coupled with 2 μg bovine serum albuin (BSA)/well. After incubation, the plates were blocked with 2% BSA in 0.05 M carbonate/bicarbonate (pH 9.6) buffer for 1 h at room temperature. Sera were diluted 50-fold in RIA buffer (1% BSA, 350 mM NaCl, 10 mM Tris-HCl, pH 7.6, 1% vol/vol Triton X-100, 0.5% wt/vol Na-deoxycholate, 0.1% SDS) supplemented with 10% fetal bovine serum; 100 μL/well were added and incubated for 1.5 h at room temperature. After washing six times with phosphate-buffered saline (PBS)/0.05% Tween-20, 100 μL/well of the anti-human secondary antibodies conjugated to peroxidase at different dilutions in RIA buffer (IgG 1:1000, IgA 1:2000, and IgM 1:40,000) were added. After incubation for 1 h at room temperature, the plates were washed six times with PBS/0.05% Tween-20 and bound antibodies were detected with o-phenylenediamine dihydrochloride (OPD; Sigma Chemical Company) and 0.8 μL/mL 30% hydrogen peroxide. The plates were incubated at room temperature for 30 min. The reaction was stopped with 50 μL of 2 N H_2_SO_4_ per well and absorbance values were measured at a wavelength of 492 nm.

All sera were tested in duplicate. Control sera were also included to monitor inter- and intra-assay variations.

### Cut-off values and specificity control

ROC curve analysis and regression analysis were conducted using the GraphPad Prism4 program and cut-off values for each test were determined with a specificity of 98% compared with a healthy population of blood donors (*n* = 64). Positive values were defined as follows: >0.188 optical density (OD) units for IgG anti-p18, >0.268 OD units for IgA anti-p18, >0.302 OD units for IgM anti-p18, >0.352 OD units for IgG anti-p48, >0.214 OD units for IgG anti-p55, >0.384 OD units for IgA anti-p55, >0.234 OD units for IgM anti-p55, >0.226 OD units for IgG anti-CEP-1, >0.810 OD units for IgA anti-CEP-1, and >0.358 OD units for IgM anti-CEP-1. RF was determined by nephelometry (NV <20 UI).

Microtiter plates coated with uncitrullinated peptides were used as a control for citrulline specificity of the anti-citrulline peptide antibodies. The OD values on the arginine variants were clearly bellow the citrulline respective peptide cut-off values. The number of patients who recognized both the citrullinated and the arginine-containing peptide with similar OD values, and thus are considered not to bind to the respective peptide in a citrulline-specific way, was very small. In this sense, only one serum of RA (4.7% of the 21 IgG anti-CEP-1-positive) and another one RA serum (3.6% of the 28 IgA anti-p18-positive sera) reacted against the respective arginine control peptides of p18 and CEP-1. Besides, for the PR sera included in this study, only one serum (5.9% of the 17 IgG anti-CEP-1-positive sera) reacted against the uncitrullinated CEP-1 peptide. These three patients were considered negative for the presence of IgG and IgA anti-CEP-1 and anti-p18 antibodies because no specific response was detectable.

### Statistical analysis

Continuous data are presented as means and standard deviation and categorical variables as absolute frequencies and percentages. Differences in demographic, clinical, and ACPA fine specificities and isotypes were compared between PR and RA patients. For continuous variables, we used the Student’s or Mann-Whitney tests. For binary variables, we used the Chi-square or Fisher’s exact tests, when appropriate. Differences in reactivity of OD values between PR and RA patients were analyzed using the Mann-Whitney test. We analyzed anti-CCP2 reactivity levels according to the number of IgG specificities using Spearman’s correlation test. The level of statistical significance was established as ≤0.05. The analysis was made using R version 3.2.0 (copyright ©2015, The R Foundation for Statistical Computing).

## Results

### Patient characteristics

We included 54 PR patients and 54 RA patients (62.9% female, 66.7% CCP2-positive in both groups). There were no significant between-group differences in mean age, disease duration, and RF positivity (Table 1). The demographic and clinical characteristics of patients with PR are described elsewhere [18].

**Table 1:** Clinical and serological characteristics of patients with palindromic rheumatism (PR) and rheumatoid arthritis (RA)

	PR (*n* = 54)	RA (*n* = 54)	*p*
Female, n (%)	34 (62.9)	34 (62.9)	NS
Age (year), mean ± SD	51.2 ± 11.3	54.7 ± 11.8	NS
Disease duration (years), mean ± SD	11.6 ± 10.7	8.3 ± 6.1	NS
RF n (%)	31 (57.4)	30 (55.6)	NS
RF levels^a^, mean ± SD	237.3 ± 300.6	379.6 ± 715.9	NS
CCP2 n (%)	36 (66.7)	36 (66.7)	NS
CCP2 levels^a^, mean ± SD	392.6 ± 527.6	487.4 ± 584.4	NS

^a^Measured only in positive patients

*CCP* cyclic citrullinated peptide, *NS* not significant, *RA* rheumatoid arthritis, *RF* Rheumatoid factor

### IgG ACPA fine specificities

PR patients had a lower frequency of some IgG ACPA fine specificities than RA patients, which was significant for the two vimentin-derived peptides, p48 vimentin (1.9% RP vs. 14.8% AR, *p* = 0.03) and, especially, p55 vimentin (PR 24.1% vs. 59.3% RA; *p* < 0.001) (Table 2). The mean number of additional ACPA specificities (p48 and p55 were grouped into one) was lower in PR patients (0.91 ± 0.96 vs. 1.43 ± 1.03; *p* = 0.008). Only 25.9% of PR patients recognized ≥2 additional IgG ACPA specificities compared with 46.3% of RA patients (*p* = 0.028) (Table 3).

**Table 2:** ACPA fine specificities (IgG, IgM, and IgA isotypes) for palindromic rheumatism (PR) and rheumatoid arthritis (RA) groups

	IgG			IgM			IgA			Any isotype [+]		
	PR (*n* = 54)	RA (*n* = 54)	*p*	PR (*n* = 54)	RA (*n* = 54)	*P*	PR (*n* = 54)	RA (*n* = 54)	*P*	PR (*n* = 54)	RA (*n* = 54)	*p*
p18 α-fibrin, *n* (%)	19 (35.2)	26 (48.1)	0.17	7 (13)	21 (38.9)	**0.02**	10 (18.5)	27 (50)	**<0.001**	28(51.9%)	37(68.5%)	0.077
p55 vimentin, *n* (%)	13 (24.1)	32 (59.3)	**<0.001**	5 (9.3)	5 (9.3)	1	9 (16.7)	21(38.9)	**0.01**	22(40.7%)	40 (74.1%)	**<0.001**
CEP-1 Enolase, *n* (%)	16 (29.6)	20 (37.0)	0.37	1 (1.8%)	0 (0)	(0.3)	1 (1.8)	3(5.6)	0.3	17(31.5%)	22(40.7%)	0.317
p48 vimentin, *n* (%)	1(1.9)	8 (14.8)	**0.03**	–	–	–	–	–	–	–	–	–

Significant *p* values are shown in bold

**Table 3:** Total number of IgG ACPA specificities in PR and RA

	PR	RA	*p*
0	23 (42.6%)	12 (22.2%)	**0.024**
1	17 (31.5%)	17 (31.5%)	1
2	10 (18.5%)	15 (27.8%)	0.25
3	4 (7.4%)	10 (18.5%)	0.08
≥2	14 (25.9%)	25 (46.3%)	**0.028**

*ACPA* anti-citrullinated peptide/protein antibodies, *PR* palindromic rheumatism, *RA* rheumatoid arthritis

Significant *p* values are shown in bold

**Fig. 1:**
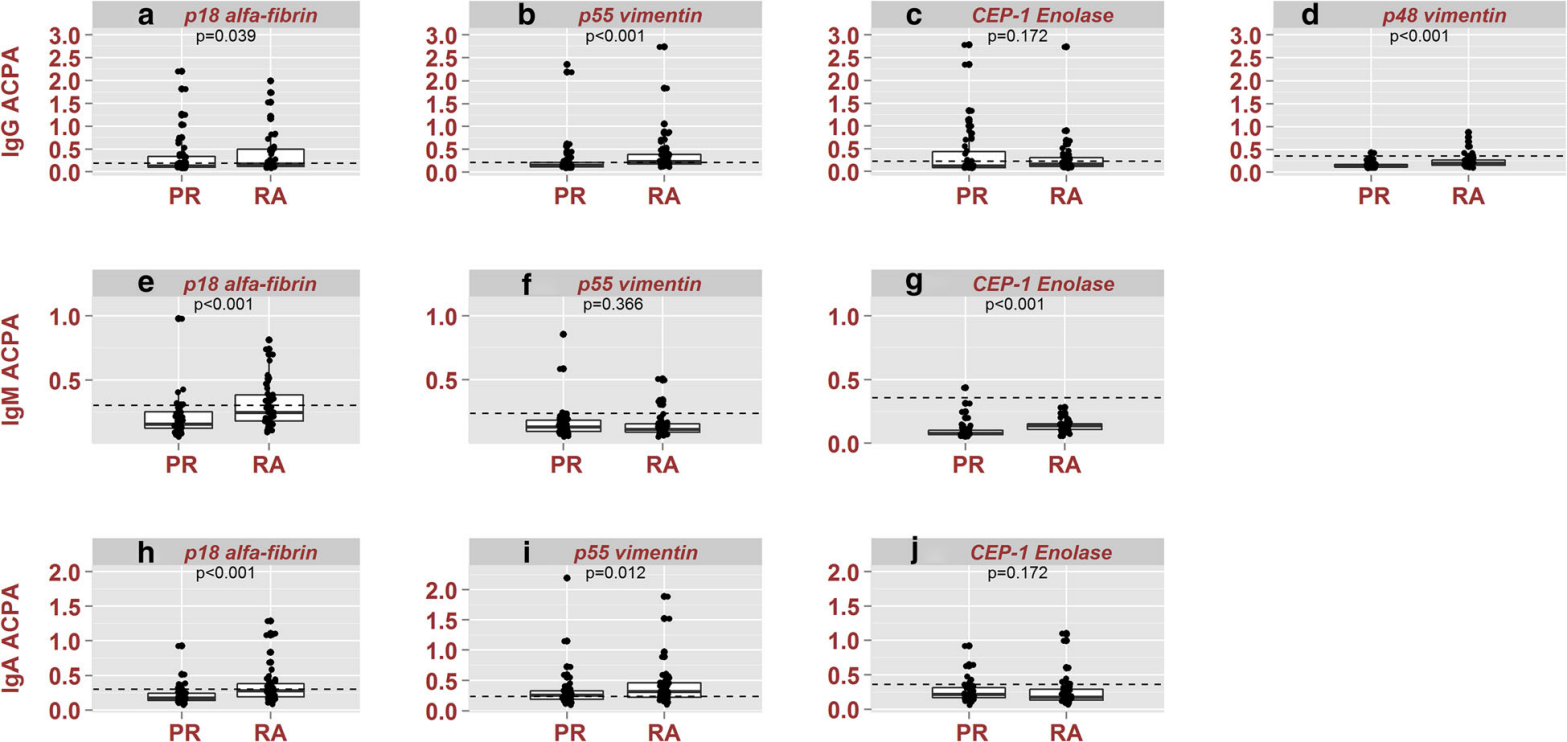
Box plots showing the levels of ACPA fine specificities and isotypes in PR. Box plots shows reactivity profiles as optical density values of anti-cyclic citrullinated peptide (*ACPA*) for IgG (**a**, **b**, **c**, **d**), IgM (**e**, **f**, **g**), and IgA (**h**, **i**, **j**) isotypes. The p18 α-fibrin, p55 vimentin, and CEP-1 enolase fine specificities were analyzed for IgG, IgM, and IgA isotypes and p48 vimentin for only IgG. ACPA reactivity profiles were compared in 54 patients with palindromic rheumatism (*PR*) and 54 patients with rheumatoid arthritis (*RA*). Mann Whitney *p* values for between-group differences were calculated. Caution should be taken with these results because OD values are related to the quantity of antibody present in a nonlinear fashion. When only patients positive (above cut-off levels) for the respective ACPA isotypes were analyzed, the *p* values for IgG p18 α-fibrin, p48 vimentin, p55 vimentin, and CEP-1 Enolase were 0.963, 0.667, 0.445, and 0.02, respectively. The *p* values for IgM p18 α-fibrin, p55 vimentin, and CEP-1 enolase were 0.126, 0.690, and not calculable (no patients [+] in the RA group), respectively. The *p* values for IgA for p18 α-fibrin, p55 vimentin, and CEP-1 enolase were 0.230, 0.929, and 0.500, respectively. Box plots show the median, percentile 25, percentile 75, minimum, and maximum. *Dots* represent the value of the observation of one patient. *Broken lines* indicate the cut-off values

Significantly higher levels of IgG ACPA fine specificities were found in RA patients compared with PR patients, except for the enolase peptide, when all samples were considered; however, no significant differences were found when negative samples were excluded except for IgG enolase, which was higher in PR (Fig. 1a, b, c, and d). Comparison of the 18 CCP2-negative patients in each group showed that p48/p55 vimentin was lower in PR patients (5.6% vs. 38.9%; *p* = 0.04) and that PR patients had a lower mean number of positive specificities (0.17 ± 0.5 PR vs. 0.56 ± 0.6 RA; *p* = 0.04).

**Fig. 2:**
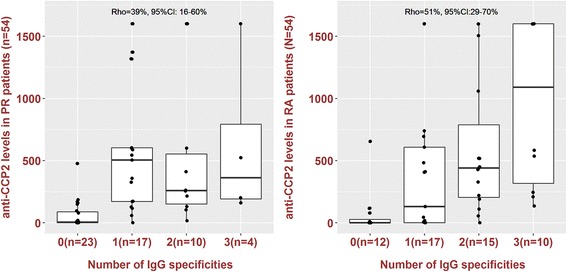
Association between the number of citrullinated epitopes recognized and CCP2 levels in palindromic rheumatism (*PR*) and rheumatoid arthritis (*RA*). Box plots show reactivity levels of CCP2 antibody according to the number of citrullinated epitopes recognized by ACPA in PR and RA patients. Spearman’s correlation coefficient (*Rho*) tested the strength of this relationship. Values of Rho between 0.4 and 0.6 suggest a moderate correlation. CCP2 reactivity levels significantly correlate with the number of citrullinated epitopes recognized by ACPA in RA, but to a lesser extent in PR. Box plots show the median, percentile 25, percentile 75, minimum,and maximum. *Dots* represent the value of the observation of one patient. *Broken lines* indicate the cut-off value. *CI* confidence interval

CCP2 antibody levels correlated with the number of citrullinated epitopes recognized by ACPA in RA, but to a lesser extent in PR (Fig. 2).

### IgA and IgM ACPA isotypes

PR patients had a lower frequency of IgA and IgM ACPA isotypes, which was significant in the case of IgA and IgM against fibrin p18 and IgA against vimentin p55 (Table 2). Mean levels of fibrin p18 IgA and IgM isotypes, enolase CEP1 IgM, and p55 vimentin IgA were lower in PR (Fig. 1e, f, g, h, i, and j). The mean number of IgA (0.37 ± 0.7 vs. 0.96 ± 0.93; *p* < 0.001) and IgM (0.22 ± 0.46 vs. 0.44 ± 0.6; *p* = 0.04) ACPA isotypes was lower in PR than in RA patients.

## Discussion

We characterized the ACPA immune response in patients with PR or established RA and found differences in the response, with a more restricted repertoire in PR patients. Most PR patients tested positive for the commercial CCP2 test, and revealed an ACPA repertoire closer to that seen in the preclinical phase of RA [9] or in unaffected relatives of RA patients than in patients with RA [12, 19, 20]. In PR patients, we found fewer ACPA fine specificities, with a significantly lower frequency of antibodies against the citrullinated peptide from vimentin and less isotype usage.

To our knowledge, this is the first study to analyze the ACPA repertoire in patients with PR. Analysis of the development of the ACPA response in the preclinical phases of RA showed that ACPA initiates in a restricted manner and expands over time until clinical synovitis is present, with no particular citrullinated peptide predominating in the early phases [9]. It is reported that, in CCP-2-positive patients with arthralgia, the ACPA profile did not differ between patients who evolved or not to overt arthritis, but patients recognizing two or more additional citrullinated peptides more frequently developed arthritis [21] and patients with undifferentiated arthritis who evolved to RA showed greater recognition of citrullinated peptides than those who did not [10]. Our results are in agreement with these findings, since the number of additional citrullinated peptides recognized by ACPA in PR patients was significantly lower than that observed in RA patients.

The low frequency of ACPA against the citrullinated peptide from p55 vimentin found in PR (24.1% vs. 59.3% in RA) is of interest. There are conflicting results on the diagnostic accuracy and prognostic significance of ACPA vimentin antibodies compared with other ACPA fine specificities in RA, probably due to the heterogeneity of the studies or methodological issues [22, 23]. Their presence in unaffected relatives of RA patients has been described as virtually absent in one study [12] whereas a prevalence of 20% was reported in another study [19].

As in RA [24], the ACPA IgG isotype predominates in PR, but our results show less ACPA isotype usage in patients with PR, with significantly less use of IgA and IgM against citrullinated fibrin peptide and IgA against citrullinated vimentin peptides. Lower frequencies of all ACPA (CCP) isotypes have been observed in patients with undifferentiated arthritis who do not evolve to RA compared with RA patients [25]. The absence of IgM against peptide p55 derived from vimentin in most patients with RA and PR (9.3% in both diseases) in our study is intriguing, since high recruitment of new B cells might be expected in RA. However, a similarly low percentage of IgM against the citrullinated peptide p55 derived from vimentin (7%) has been found in patients with RA [26].

This lower frequency of isotype usage and fine citrullinated specificities in PR was observed even when the level of CCP2 did not differ significantly between PR and RA. We and others have observed that levels of anti-CCP in PR were similar to those seen in RA [2, 3]. An association between anti-CCP2 levels and the number of citrullinated peptides recognized has been described in RA using an ELISA test [27] or microarray techniques [27–29], and was observed in RA patients in the present study; however, we found that this association was observed to a lesser extent in PR, although the low number of recognized epitopes in PR patients limits data interpretation. Previous studies in patients with ACPA-positive arthralgia showed a higher risk of RA progression in patients with high levels of anti-CCP [30], but in the study of Verpoort et al. [26], although a trend toward high levels of anti-CCP was observed in patients with CCP-positive RA compared to CCP-positive undifferentiated arthritis for all isotypes, no significant differences were observed when negative samples were excluded. The authors concluded that the level of the different isotypes did not differ between RA and undifferentiated arthritis, but the number of antibody-positive patients was higher in RA than in undifferentiated arthritis. Similar results were observed in our study.

One limitation of this study is the selection bias towards a more stable population of longstanding PR patients who are less prone to evolve to RA; thus, the findings cannot be extrapolated to PR patients with a short disease duration. The strengths of the study include the strict selection criteria for PR, excluding other causes of arthritis and the presence of persistent arthritis in these patients, and an RA patient population controlled for disease duration and ACPA positivity by the CCP2 test. Likewise, the high accuracy of home-made ELISA tests based on citrullinated synthetic peptides in the diagnosis of RA has been shown in previous studies.

## Conclusions

We observed a more restricted pattern of ACPA recognition in patients with longstanding PR, with fewer fine specificities (especially in the case of the peptide of citrullinated vimentin) and lower isotype usage than in RA patients, an ACPA repertoire most frequently reported in the preclinical phase of RA or unaffected relatives of RA patients. It may be speculated that some patients with PR who do not evolve to RA may have impaired maturation of the B-cell response against citrullinated peptides that may stop the development of chronic arthritis. Prospective studies in the early phases of PR are warranted.

## Abbreviations

ACPA: Anti-citrullinated peptide/protein antibody
CEP-1: [Cys^4,22^,Cit^9,15^]α-Enolase(5-21)
ELISA: Enzyme-linked immunosorbent assay
OD: Optical density
p18: [Cit^630^]α-Fibrin(617-631)
p48: [Cit^71^]Vim(47-72)
p55: [Cit ^64,69,71^]Vim (47-72)
PR: Palindromic rheumatism
RA: Rheumatoid arthritis
RF: Rheumatoid factor
ROC: Receiver operating characteristic
